# Surveillance of Arthropod-Borne Viruses and Their Vectors in the Mediterranean and Black Sea Regions Within the MediLabSecure Network

**DOI:** 10.1007/s40475-017-0101-y

**Published:** 2017-03-17

**Authors:** Anna-Bella Failloux, Ali Bouattour, Chafika Faraj, Filiz Gunay, Nabil Haddad, Zoubir Harrat, Elizabeta Jancheska, Khalil Kanani, Mohamed Amin Kenawy, Majlinda Kota, Igor Pajovic, Lusine Paronyan, Dusan Petric, Mhammed Sarih, Samir Sawalha, Taher Shaibi, Kurtesh Sherifi, Tatiana Sulesco, Enkelejda Velo, Lobna Gaayeb, Kathleen Victoir, Vincent Robert

**Affiliations:** 1 Department of Virology, Arboviruses and Insect Vectors, Institut Pasteur, Paris, France; 2 Laboratory of Medical Entomology, Institut Pasteur of Tunis, Tunis, Tunisia; 3 Laboratory of Medical Entomology, Institut National d’Hygiène, Rabat, Morocco; 4 Hacettepe University, HU-ESRL-VERG, Ankara, Turkey; 5 Faculty of Public Health, Laboratory of Immunology, Lebanese University, Beirut, Lebanon; 6 Eco-Epidemiologie Parasitaire et Génétique des Populations, Institut Pasteur of Algeria, Alger, Algeria; 7 Laboratory for Virology and Molecular Diagnostics, Institute of Public Health, Skopje, Macedonia; 8 Parasitic and Zoonotic Diseases Department, Ministry of Health, Amman, Jordan; 9 Department of Entomology, Faculty of Science, Ain Shams University, Cairo, Egypt; 10 Department of Control of Infectious Diseases, Laboratory of Virology, Institute of Public Health, Tirana, Albania; 11 Biotechnical Faculty, Laboratory for Applied Zoology, University of Montenegro, Podgorica, Montenegro; 12 Vector Borne and Parasitic Diseases Epidemiology Department, National Center for Diseases Control and Prevention, Yerevan, Armenia; 13 Faculty of Agriculture, Laboratory of Medical and Veterinary Entomology, University of Novi Sad, Novi Sad, Serbia; 14 Laboratory of Vectorial Diseases, Institut Pasteur of Morocco, Casablanca, Morocco; 15 Laboratory of Public Health, Ministry of Health, Ramallah, Palestine; 16 Laboratory of Parasitology and Vector-Borne Diseases, National Center for Disease Control, Tripoli, Libya; 17 Faculty of Agriculture and Veterinary Science, Institute of Veterinary Medicine, University of Prishtina, Prishtina, Kosovo; 18 Laboratory of Systematics and Molecular Phylogeny, Institute of zoology, Chisinau, Republic of Moldova; 19 Department of Control of Infectious Diseases, Vector Control Unit, Laboratory of Medical Entomology, Institute of Public Health, Tirana, Albania; 20 Department of International Affairs, Institut Pasteur, Paris, France; 21 French National Research Institute for Sustainable Development, MIVEGEC Unit, IRD224-CNRS 5290-Montpellier University, Montpellier, France

**Keywords:** Arboviruses, Vectors, Emergence, Surveillance, Mediterranean and Black Sea regions

## Abstract

**Purpose of Review:**

Arboviruses, viruses transmitted by arthropods such as mosquitoes, ticks, sandflies, and fleas are a significant threat to public health because of their epidemic and zoonotic potential. The geographical distribution of mosquito-borne diseases such as West Nile (WN), Rift Valley fever (RVF), Dengue, Chikungunya, and Zika has expanded over the last decades. Countries of the Mediterranean and Black Sea regions are not spared. Outbreaks of WN are repeatedly reported in the Mediterranean basin. Human cases of RVF were reported at the southern borders of the Maghreb region. For this reason, establishing the basis for the research to understand the potential for the future emergence of these and other arboviruses and their expansion into new geographic areas became a public health priority. In this context, the European network “MediLabSecure” gathering laboratories in 19 non-EU countries from the Mediterranean and Black Sea regions seeks to improve the surveillance (of animals, humans, and vectors) by reinforcing capacity building and harmonizing national surveillance systems to address this important human and veterinary health issue. The aim of this review is to give an exhaustive overview of arboviruses and their vectors in the region.

**Recent Findings:**

The data presented underline the importance of surveillance in the implementation of more adapted control strategies to combat vector-borne diseases. Partner laboratories within the MediLabSecure network present a wide range of infrastructures and have benefited from different training programs.

**Summary:**

Although reporting of arboviral presence is not carried out in a systematic manner, the expansion of the area where arboviruses are present cannot be disputed. This reinforces the need for increasing surveillance capacity building in this region to prevent future emergences.

## Introduction

The global distribution and disease burden associated to arboviruses have increased over recent years. Unexpectedly, Chikungunya hit northeastern Italy in 2007 and struck France in 2010 and 2014 [1]. Dengue entered the European scene in 2010 followed by more human cases in subsequent years [2]. A more recent threat is the Zika virus (ZIKV) which is still unreported in the European territory, including the Mediterranean region, despite the increasing number of imported cases [3, 4••].

In countries of the Mediterranean and Black Sea regions, the epidemiology (presence, risk, transmission, etc.) of arboviruses remains poorly characterized. MediLabSecure is a European project (2014–2018) that has established a laboratory network including partners from 19 countries of the Mediterranean and Black Sea regions. These countries are in the Balkans, around the Black Sea, in South Caucasus, Middle East and North Africa. As they share common public health issues and threats, the overall objective of MediLabSecure is to increase through capacity building and harmonization of surveillance systems the health security in the whole Mediterranean region. Emerging mosquito-borne viruses that are pathogens for humans and/or animals are at the heart of this project: West Nile (WN), Dengue (DEN), Chikungunya (CHIK), and Rift Valley Fever (RVF). Within this One Health network, a subset of laboratories are dedicated to medical and veterinary entomology, while others are focused on human virology, animal virology, and public health. The entomologists target mosquitoes identified as present threats or with potential risk of emergence in the concerned region: *Aedes* mosquitoes (*Aedes aegypti* (=*Stegomyia aegypti*) and *Aedes albopictus* (=*Stegomyia albopicta*)) responsible for transmission of Dengue virus (DENV) and Chikungunya virus (CHIKV) to humans and *Culex* mosquitoes (e.g., *Culex pipiens*) implicated in the transmission of West Nile virus (WNV). Other arboviruses transmitted by sandflies or ticks (e.g., Crimean-Congo hemorrhagic fever (CCHF) virus transmitted by *Hyalomma* tick bites) are also considered in the MediLabSecure program activities.

## The Past of Mosquito-Borne Diseases in Europe

Dengue (DEN) was not uncommon in the Mediterranean area in the past. The disease was reported in Athens in 1928 which was the theater of the last major epidemic on the European continent with 1 million cases and 1000 deaths [5]. It seemed that clinical severity was due to the sequential circulation of viruses DENV-1 and DENV-2. *Aedes aegypti*, the vector of dengue in Greece, disappeared from the Eastern Mediterranean after 1935 [6]. Since then, no local transmission of Dengue has been recorded in Europe until 2010 when autochthonous cases of Dengue were reported in Croatia and France [2].

Yellow Fever (YF) is mainly transmitted to humans by *Ae. aegypti*. While originating from Africa, YF was first reported in the Caribbean after the introduction of its vector via the slave trade from West Africa. Yellow Fever and *Ae. aegypti* were introduced around 1700 in harbors of Western Europe. Outbreaks occurred on the Iberian Peninsula (Gibraltar, Sevilla, Cadiz, Malaga, Lisbon, Porto, Barcelona), France (Marseille, Brest, Saint-Nazaire, Rochefort, Bordeaux), and Italy (Livorno) during the nineteenth and beginning of the twentieth centuries [7]. More than 5000 people died in Barcelona between 1821 and 1824, and more than 6000 in Lisbon in 1857 [8].

West Nile (WN) has been discovered in Africa in 1937 and circulated on the continent mainly associated with mild symptoms. Since the 1990s, new viral strains were responsible for increased incidence of human diseases in Europe. While lineage 1 was repeatedly isolated in Europe and North Africa, lineage 2, historically endemic in sub-Saharan Africa and Madagascar, has been recently associated with human cases in Hungary with subsequent spread into Austria, Italy, Russia, Greece, Serbia, and Croatia [9]. Currently, WNV is present in Africa, the Middle East, Europe, Asia, America and has become the most widely distributed of the encephalitic flaviviruses. Contrary to related flaviviruses such as DENV and YFV, WNV can be transmitted by wide range of vector species and has been detected in more than 59 different mosquito species [10].

## The Vectors of Arboviruses in Europe

***Aedes aegypti***: This mosquito was common in southern Europe and the Middle East at the beginning of twentieth century [6, 11]. It was present in southern Europe and Western Asia at the beginning of twentieth century, in Syria, Lebanon, Turkey, Greece, former Yugoslavia, Italy, Corsica (France), and Spain [6, 11]. The species was the vector of a YF outbreak in Livorno in Italy in 1804. Following the development of sanitation and management of urban water collections and anti-malaria vector control with DDT, the vector disappeared from continental Europe after the 1950s. The last record of *Ae. aegypti* in Europe was in Desenzano del Garda (Brescia province, northern Italy) in 1971 [12] . An *Ae. Aegypti*-vectored outbreak was reported in Madeira in 2012 [13]. After decades of absence, *Ae. aegypti* is back to the Eurasian continent, more specifically around the Black Sea in southern Russia, Apkhazeti, and Georgia in 2004 [14], and north-eastern Turkey [15••]. Major outbreaks associated with *Ae. aegypti* may occur in South Europe as this mosquito was responsible for large epidemics of Dengue in Greece in 1927–1928 [5]. Additionally, this species is a vector of CHIKV and ZIKV.

***Aedes albopictus***: From its native home-range in the forests of South-East Asia, *Ae. albopictus* [16] has succeeded in colonizing most continents in the past 30–40 years [17]. The species was recorded for the first time in Europe in Albania in 1979 [18], then in Italy in 1990 [19, 20], and is now present in 20 European countries [21]. Today, it is established in most countries of the Mediterranean Sea, including Lebanon, Syria, and Israel. This mosquito is susceptible to 26 arboviruses including CHIKV, DENV, and YFV when provided by experimental infections [22]. Unexpectedly, *Ae. albopictus* was responsible for CHIKV and DENV cases in Europe. Chikungunya virus emerged in Europe: in Italy in 2007 [23] and in France in 2010 [24] and 2014 [25]. Dengue virus was detected in patients in Croatia in 2010 [26] and in France in 2010 [27], 2013 [28], 2015 [29].

***Culex pipiens***: *Cx. pipiens* complex should be considered as a polytypic species differentiated into several forms. The species *Cx. pipiens* is commonly found in the Palearctic and Oriental regions and described under two morphologically identical biotypes, named *pipiens* (Linnaeus 1758) and *molestus* (Forskål 1775), which show distinct feeding behavior. Biotype *pipiens* prefers birds as blood hosts whereas the biotype *molestus* prefers mammals [30]. Hybrids between the two biotypes have an intermediate host preference, which makes them ideal vectors to bridge arboviruses such as WN and Usutu from birds to mammals [31, 32]. The epidemiological cycle of WN disease involves migratory birds acting as reservoir and ornithophilic *Culex* mosquitoes mainly as vectors amplifying viral traffic between bird populations [33]. Migratory birds ensure the introduction of the virus from Africa into temperate areas, North Africa and Europe [34, 35]. Humans and horses present clinical symptoms, sometimes severe, but are generally considered dead-end hosts, as the level of viremia they develop is not high enough to infect mosquitoes.

Ticks: In Europe, ticks are the most important vectors of human and animal pathogens. They transmit several viruses such as tick-borne encephalitis virus (TBEV) and Crimean-Congo hemorrhagic fever virus (CCHFV) that are reemerging in many parts of the world. Tick-borne viruses (TBV) are traditionally maintained in a natural cycle between vector ticks and wild animal hosts, humans being accidental hosts. New TBVs are continually being discovered [36] presumably resulting from the proliferation of ticks in many regions of the world and incursion of humans into tick-infested habitats. *Ixodes ricinus* is the most widespread and abundant European tick presenting a high potential as vector of different pathogens: *Borrelia* bacteria responsible for Lyme disease, *Anaplasma* spp., *Rickettsia*, *Babesia,* and *Theileria. Hyalomma* ticks are aggressive species, which search for humans actively. *Hyalomma marginatum* and *Hyalomma anatolicum* are the main vectors of CCHF [37]. Other species of *Rhipicephalus*, *Boophilus*, *Haemaphysalis*, *Amblyomma*, *Dermacentor*, and *Hyalomma* genera play a role in maintaining enzootic circulation of CCHFV among tick vectors and wild/domestic mammals [37].

## The Situation of Arboviruses in Countries Around the Mediterranean and Black Sea Regions (See Fig. 1 and Table 1) (in Alphabetical Order)

**Fig. 1:**
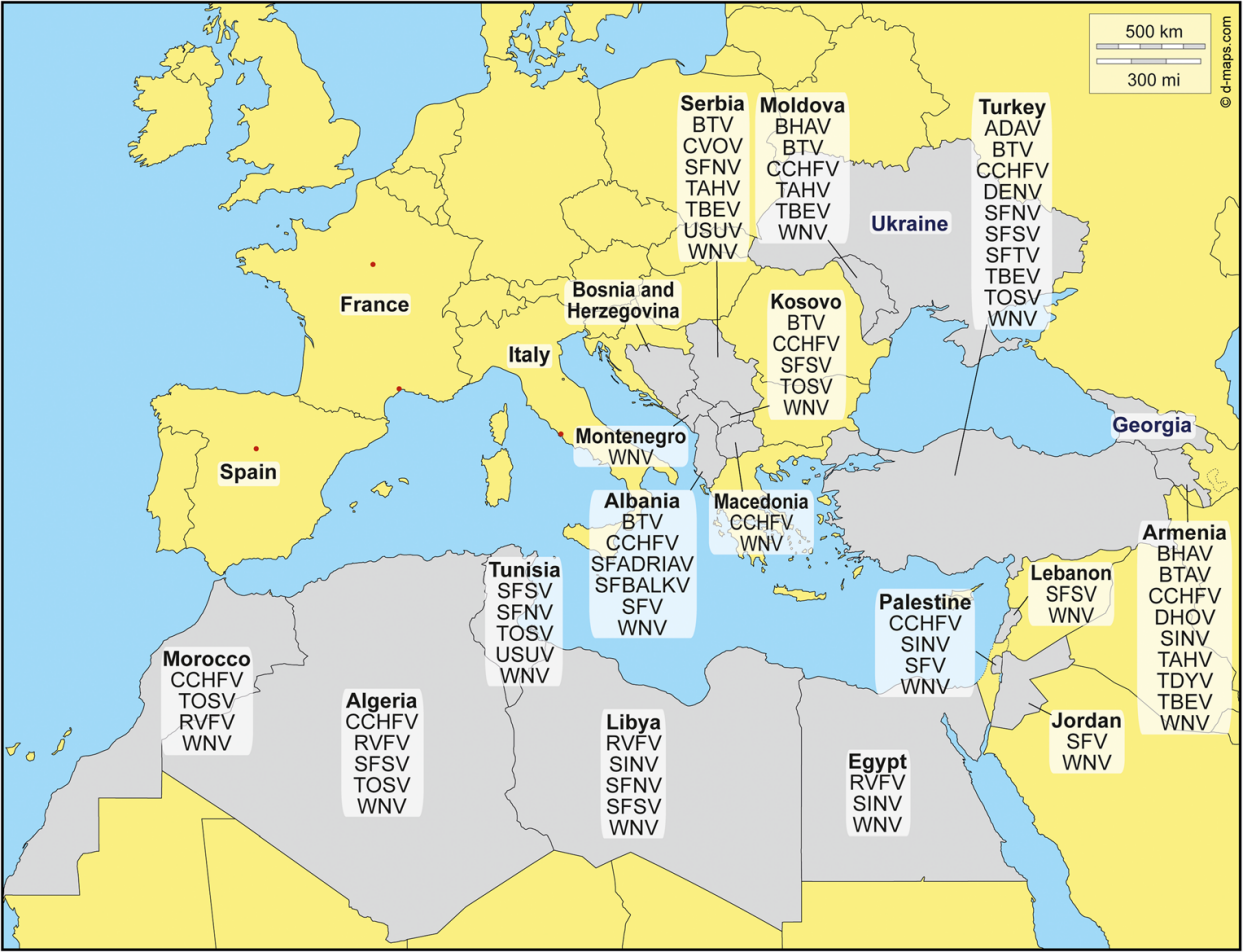
Map showing the arboviruses detected in countries that are partners of the MediLabsecure network

**Table 1:** Potential mosquito vectors of arboviruses in 16 non-EU countries from the Mediterranean and Black Sea regions participating to the MediLabsecure network

Genus	Sub-genus	Species	Albania	Algeria	Armenia	Bosnia and Herzegovine	Egypt	Georgia	Jordan	Lebanon	Libya	Rep. of Macedonia	Moldova	Morocco	Montenegro	Palestine	Serbia	Tunisia	Turkey	Ukraine	Total
*Aedes*	*Stegomyia*	*aegypti*					1	1											1		3
*Aedes*	*Stegomyia*	*albopictus*	1	1	1	1		1	1	1				1	1	1	1		1		12
*Aedes*	*Ochlerotatus*	*caspius*	1	1	1	1	1	1	1	1	1	1	1	1	1	1	1	1	1	1	18
*Aedes*	*Aedimorphus*	*vexans*	1	1	1	1		1			1	1	1	1	1		1	1	1	1	14
*Culex*	*Barraudius*	*modestus*	1	1				1				1	1	1	1		1		1	1	10
*Culex*	*Culex*	*perexiguus*	1	1			1		1	1	1	1		1		1	1	1	1		12
*Culex*	*Culex*	*pipiens*	1	1	1	1	1	1	1	1	1	1	1	1	1	1	1	1	1	1	18

### Albania

Former studies on the phlebotomine fauna of Albania showed that eight species are present: *Phlebotomus neglectus*, *Phlebotomus papatasi*, *Phlebotomus perfiliewi*, *Phlebotomus tobbi*, *Phlebotomus similis*, *Phlebotomus simici*, *Sergentomyia dentata* and *Sergentomyia minuta*, with *P. neglectus* being the most abundant and widespread species in Albania [38, 39]. A new phlebovirus, provisionally named Adria virus has been detected in 2/12 pools of sandflies trapped close to the Adriatic Sea (Kruje and Lezhe). This new virus is genetically close to Arbia virus (similarity 77.1% at nucleotide level), which belongs to the Salehabad serocomplex. Its distribution and probable pathogenicity to humans are still unknown [40]. A new virus has been detected by a next-generation sequencing approach in sandflies of Kruje region in 2014; it has been named Balkan virus belonging to the group of Sandfly fever Naples virus [41]. The Balkan virus is closely related to the Tehran virus with 16 and 3% of divergence at nucleotide and amino acid levels, respectively.

Crimean-Congo hemorrhagic fever virus (CCHFV) is endemic in Albania with intermittent outbreaks. The first CCHF case was described in 1986 in the northern part of the country (Kukes) near the border with Kosovo. Reemergence in the southern part of the country occurred in 2011 (Korce area, bordering Greece). Ticks collected during the 2003–2005 period from cattle were tested for presence of CCHFV RNA, while serum samples collected from goats, cattle, hares, and birds were tested for the presence of specific IgG antibodies to CCHFV. One of the 31 pools of ticks, consisting of four female *Hyalomma* spp. ticks, was found to carry CCHFV RNA with 99.2–100% homology to sequences detected in patients from the same region. Antibodies were not detected in cattle, hares, and birds, but two goats (among 10) presented high titers of IgG antibodies. The shepherd of the flock was a member of a family affected by CCHF 10 days before the collection of goats’sera, and he presented a mild form of the disease [42]. The overall CCHFV antibody seroprevalence rate for ruminants in Albania was 23% [43].

In 1958, WN antibodies were detected in two human blood samples [44]. In 2011, confirmed cases of WNV infections have been reported in humans. They were located in the coastal and in the central parts of Albania [45].

### Algeria

West Nile virus (WNV) has been first isolated in Algeria in 1968 from a pool of *Culex* mosquitoes in the district of Djanet, a Saharan oasis in the southeastern part of the country [46]. In the 1970s (1973, 1975, and 1976), several serological surveys revealed WNV circulation in human populations in the Sahara and steppe areas [47]. In 1994, more than 50 human cases and 8 fatalities were documented during an outbreak of WNV encephalitis in Tinerkouk oasis in the south-west of Algeria [48]. In 2012, a retrospective serosurvey in Algiers and surrounding areas highlighted specific anti-WNV IgG in 11 out of 164 human samples tested [49]. During the same year, a fatal neuro-invasive case was reported for the first time in northern Algeria, in the province of Jijel [50]. In 2013, two more cases were notified, in the province of Guelma and in the Sahara desert, in the province of Timimoune [51]. Recently, the WNV lineage 1 was detected in a pool of *Culex perexiguus* collected from the Oasis of Aougrout, province of Timimoune (unpublished data).

Among sandfly-borne phleboviruses reported in Algeria, the Sandfly Fever Sicilian virus (SFSV) has been detected both in humans and sandflies in Tizi Ouzou district, northern part of the country [52]. Between 2015 and 2016, serological evidence of Toscana virus (TOSV) circulation was reported both in humans and dogs [53, 54]. During the same period, TOSV was isolated from sandflies collected in Tizi Ouzou [53]. Furthermore, several cases of meningitis and meningo-encephalitis due to TOSV were detected these last 2 years (unpublished data).

Regarding Rift Valley fever (RVF), Algeria as well as all North Africa region is at high risk with respect to the epidemiological situation in neighboring countries in the Sahara, with recurrent outbreaks in Mauritania (2010, 2012 and 2015) and recently in Niger (2016). So far, there has been no human case reported in Algeria, despite evidence from serological data that the virus circulated in the southern part of the country [55].

Between 2015 and 2016, only few imported cases of dengue were reported (unpublished data); however, the risk of autochthonous cases remains significant since *Ae. albopictus*, the vector of DENV, CHIKV, and ZIKV, has been recently established in two high densely populated cities in Algeria, Oran [56] and Algiers (unpublished data). Earlier this year 2016, the Crimean-Congo Hemorrhagic fever virus (CCHFV) has been isolated from *Hyalomma aegyptium* ticks in the South of Algeria [57].

### Armenia

Armenia is located in the South Caucasus and is mainly composed of highlands and mountains. Until now, five genera of Culicidae have been described in Armenia. The genus *Anopheles* is represented by six species *An. maculipennis*, *An. sacharovi*, *An. claviger*, *An. hyrcanus*, *An. superpictus*, *An. plumbeus* [58]. The most common Culicine species are: *Cx. pipiens*, *Cx. hortensis*, *Cx. theileri*, and *Ae. caspius*. In 2006, an extensive entomological survey has allowed to collect 64,567 mosquitoes and 45,180 *Ixodidae* ticks and identify 125 distinct strains of arboviruses in four climatic regions: the dry steppe (up to 800 m above sea level), desert and semi-desert (800–1100 m above sea level), mountain steppe, mountain forest (2000–2500 m) and Alpine (above 2500 m). From the 64,567 mosquitoes, 47,674 were *Anopheles* (73.8%), 11,252 *Culex* (17.4%) and 5641 *Aedes* (8.7%). From 45,180 ticks are found 31,371 *Dermacentor* (70%), 4425 *Rhipicephalus* (10%), 3630 *Hyalomma* (8%), 2913 *Boophilus* (6%), 1747 *Haemaphisalis* (4%), and 1094 *Ixodes* (2%). From mosquitoes, four viruses have been isolated: Batai (*Orthobunyavirus*, Bunyaviridae) in *Culex* mosquitoes, Sindbis (*Alphavirus*, Togaviridae) in *Culex*, Tahyna (*Orthobunyavirus*, Bunyaviridae) in *Aedes* mosquitoes, and WNV in *Cx. pipiens*. From ticks, at least five viruses have been detected: TBEV (*Flavivirus*, Flaviviridae) in *Ixodes*, Dhori (*Thogotovirus*, Orthomyxoviridae) in Ixodidae, Bhanja (Phlebovirus, Bunyaviridae) in *Haemaphysalis*, Tamdy (*Nairovirus*, Bunyaviridae) in Ixodidae, and CCFHV (*Nairovirus*, Bunyaviridae) in *Hyalomma* [59].

### Lebanon

Lebanon is located on the eastern side of the Mediterranean Sea that is considered endemic for several arboviral diseases such as West Nile that has been reported from neighboring countries: Israel [60] and Turkey [61]. A number of epidemics of Dengue fever had occurred in Lebanon in the past, of which one was reported from Beirut region and affected more than 100,000 individuals in 1945–1946. A serological survey conducted in 1962 to 1963 using the hemagglutination-inhibition test showed a seroprevalence for WNV and DENV of 62.5 and 61.9%, respectively. During the malaria control program and as a result of mosquito control efforts, *Ae. aegypti*, the widespread dengue vector, was eliminated from the country. With the beginning of the civil war in 1975, all vector control programs ceased and never resumed since then. Consequently, mosquito populations became a public health burden and a source of nuisance. Entomological studies conducted in 2002 showed a widespread of *Cx. pipiens* in the country in addition to the presence of other arbovirus vectors such as *Cx. perexiguus*, a species involved in the transmission of WNV in Israel [62]. Moreover, *Ae. albopictus* was introduced to Lebanon and recorded for the first time in 2002 [63]. This mosquito is now established in the country and is widespread in the coastal areas around big cities and in middle altitude humid regions. Local strains of the Asian tiger mosquito showed to be competent mainly for transmission of CHIKV but also DENV [64]. In 2007, an outbreak of Sandfly fever virus occurred in Nahr El Bared, near Tripoli, North Lebanon. More than 700 cases were declared among Lebanese soldiers during military operations. A Sandfly fever virus strain close to the Sicilian strain was identified as the causative agent (Lebanese Ministry of Health sources, unpublished data). Tick-borne viruses have never been documented in Lebanon. Some of these viruses such as CCHFV occur in some neighboring countries: Turkey and Iran [65, 66]. Presently, Lebanon is considered at risk for the occurrence of several arboviral diseases such as WNV. Not only is Lebanon situated in an endemic area, but it also constitutes a major stopover for migrating birds, WNV principal reservoir. *Aedes*-borne viruses, such as CHIKV, DENV, and ZIKV, should also be regarded as potential threats. Their introduction to the country is highly likely if we consider the important flux of Lebanese expatriates coming from endemic areas in Latin America, Africa, and South-East Asia.

### Libya

Libya has become a place of passage for migrants who cross the country to Europe. They mainly come from Sub-Saharan Africa, where several arboviral diseases are endemic. As well, the illegal animal trade can be a risk for the introduction of zoonotic viruses such as RVFV. Libya is then subjected to regular serological surveys for arbovirus antibodies [67, 68]. In the most recent survey, 950 human blood samples collected in the country were examined to determine the seroprevalence (baseline exposure) to zoonotic viruses and bacteria causing acute febrile illness [68]. Antibodies against WNV (13.1%), SFNV (0.5%), SFSV (0.7%), Sindbis virus (SINV, 0.5%), and RVFV (0.4%) have been detected. For WNV, the prevalence among the Tripoli population is 2.8% (unpublished data) and 25% of the population in the Yfran area for TOSV (unpublished data).

### The Former Yugoslav Republic of Macedonia

In the Republic of Macedonia, CCHF and WN are mandatory notifiable diseases. In 1970, the first outbreak of CCHF occurred in the Ciflik village, Tetovo, with 13 confirmed cases, of which there were two deaths. Until 2002, 11 new cases of CCHF were notified. During the period 2002–2010, only one new case was registered. From a total of 128 ticks collected from cattle, sheep, and goat, the ratio between *Hyalomma* and *Rhipicephalus* ticks was 1:3. Between 2009 and 2011, a seroepidemiological study for CCHFV on 158 serum samples collected from cattle, the prevalence of antibodies rated up to 80% in the cattle population from the Northeastern region of Macedonia [69].

Serological investigation on human for WNV IgM and IgG was performed since 2010 by ELISA. Until 2016, 214 human cases with symptoms of neurologic illness were tested: 53 were positive with WNV IgM antibodies.

### Moldova

WNV is the main arbovirus isolated in Moldova. It has been detected in ticks in central and southern regions of Moldova between 1974 and 1978: *Ixodes ricinus* (1974, 1975) and *Dermacentor marginatus* (1974, 1976 and 1978) from Nisporeni, Hincesti, and Vulcanesti regions. CCHFV has been isolated from *I. ricinus*, *D. marginatus*, and *Haemaphysalis punctata* between 1973 and 1974 from Slobozia, Nisporeni, Hincesti, and Ceadir-Lunga regions [70]. TBEV was isolated from *D. marginatus* in 1975 in Nisporeni region and from mosquitoes in Moldova [71]. Batai virus has been detected in *An. maculipennis* s.l. in 1977 in Zberoaia village (western Moldova) [72]. Tahyna virus and WNV were isolated from mosquitoes in Moldova [71, 73]. Recent epidemiological studies in Romania in the Danube Delta, close to the border to Moldova reported the presence of WNV lineage 2 in mosquitoes and Ixodidae ticks collected from migratory birds [74, 75]. Migratory birds are well-known reservoir hosts for a number of arboviruses and play an important role in distributing pathogens within and between countries [76]. The Danube Delta is one of the most important migratory sites in Europe and the risk of arbovirus dissemination through the birds to Moldova is extremely high.

### Montenegro

Using different trapping methods of adults and immature stages, 174 sampling sites have been examined during 127 sampling nights in 20 of 22 municipalities in Montenegro. A total of 22 mosquito species were identified: 3 *Anopheles* potential vectors of malaria (*An. saccharovi*, *An. maculipennis*, *An. plumbeus*), 12 *Aedes* with *Aedes vexans* and *Ae. caspius*, potential vector of RVFV, *Ae. albopictus* (vector of CHIKV and DENV), 3 *Culex* with *Cx. modestus* and *Cx. pipiens*, potential vector of WNV and RVFV, 2 *Cs*. (*Culiseta annulata*, *Cs. longiareolata*), and 2 *Coquillettidia* (*Cq. richiardii* and *Cq. buxtoni*) (study done by the LOVCEN project in cooperation with the MediLabSecure and VectorNet projects). Montenegro is a country susceptible to be hit by a mosquito-borne viral disease as its neighboring country Croatia which experienced in 2010 an autochthonous transmission of DENV [26].

### Morocco

The most prevalent arbovirus in Morocco is WNV, mainly affecting horses with epizootics detected in 1996 [77], 2003 [78], and 2010 [79]. The viral isolates from 1996 to 2003 belonged to WNV lineage 1, clade 1a. In 1996, WNV infection was first detected in a patient [77]. In 2008, a serosurvey of wild birds confirmed the circulation of WNV in native birds [80]. Serological surveillance confirmed that WNV is widely present [81]. *Culex pipiens* is highly suspected in the transmission of WNV in Morocco [82]. Moreover, RVF is another arbovirus also transmitted by *Cx. pipiens*, which circulates at a common border with Mauritania where the first West African outbreak of RVF occurred in 1987 [83]. Following this epidemic, Mauritania has experienced several other episodes: in 1993, 1998, 2003, 2010, and 2012 [84, 85]. In 2010, following unusual heavy rains, an epidemic affecting mainly camels presenting severe clinical signs and high mortalities was reported in northern Mauritania. It has been suggested that the oasis can play a role as a site of emergence of RVF in the Maghreb where eco-climatic and entomological conditions are suitable for vector development. The border with Mauritania consists of hundreds of kilometers which are a real strainer to unauthorized migrants, nomads and domestic animals. Serological surveys of humans and animals should be reinforced in addition to viral isolations in mosquitoes. Apart from its role as vector, *Cx. pipiens* is mainly considered a nuisance in most urban areas. It is for this reason that it has been targeted for years in mosquito control programs. The most widely used insecticides are pyrethroids, organophosphorus and carbamates. Although the level of resistance to insecticides of this mosquito is the object of a regular surveillance by the Ministry of Health since 2003, published data remain fragmentary. Overall, *Cx. pipiens* populations showed significant resistance to insecticides used [86, 87] compromising the strategy of mosquito control. Other arboviruses such as DENV, CHIKV and ZIKV were not yet detected in Morocco. However, the vector *Ae. albopictus* has been detected recently in the town of Rabat [88].

Regarding sandfly-borne viruses, TOSV is known to be present in Morocco [89, 90]. It was isolated from *P. sergenti* and *P. longicupis* collected in north and center of Morocco between 2008 and 2011 [91]. Moreover, CCHFV has been isolated from *Hyalomma marginatum* ticks in South Morocco in 2011 [92].

### Palestine

Only sporadic cases of WN have been detected in Palestine. Since 2000, six human cases were reported in the West Bank of Palestine with four cases occurring in the northern districts (2 cases in 2000 in Nablus and Jenin districts, and 2 cases in 2008–2010 in Qalqilia district) and two cases in Jericho city in 2011–2014. All patients were males between 35 and 65 years old. One patient from Jericho died from the WNV infection. *Cx. pipiens* was suspected as the main vector; it is present in the West Bank, especially in Tubas, Qalqiliah, Jericho, and Bethlehem districts and active all year long with the highest densities in summer. Other arboviral diseases are occasionally detected: CCHF, SINV fever, and Sandfly fever. In Palestine, arboviral diseases must be notified to the Preventive Medicine Division in the district of the patient’s residence. Then a special investigation is set off with reports to the Central Preventive Medicine Department. Control measures are immediately implemented by the vector control unit of the Health Promotion Department. In addition to *Cx. pipiens*, three other mosquito species were reported: *Ae. albopictus*, *An. claviger*, and *Cs. longiareolata. Aedes albopictus* widely found in the West Bank (Tubas, Qalqiliah, Jericho and Bethlehem districts), is active from March to November with the highest densities reported in August. Monthly investigations at district level for breeding sites are implemented by the vector control unit. In 2015, about 7500 sites were inspected for mosquito breeding and 61% were positive for immature stages. Eighty-five percent of positive sites were treated using environmental measures and 9% treated with pesticides (pyrethroids or *Bacillus thuringiensis*).

### Serbia

Serbia experienced the second largest outbreak of WN in Europe, with 200 confirmed human cases in 2013 (http://ecdc.europa.eu/en/healthtopics/west_nile_fever/West-Nile-fever-maps/Pages/historical-data.aspx#sthash.S97C32Ep.dpuf). The largest numbers of human cases, as well as outbreaks of various magnitudes, have been reported repeatedly in the Vojvodina province of northern Serbia since 2012 (9 in 2012, 85 in 2013, 27 in 2014, and 10 in 2015) (http://www.batut.org.rs/index.php). The first serological investigation for WNV was conducted in 1972, and antibodies against WNV were found in 2.6–4.7% of human sera [93]. Between 2001 and 2005, the seroprevalence for WNV was 6.7% in 45 patients who had been hospitalized for encephalitis or meningoencephalitis. Information on asymptomatic cases was also provided: 3.7% among 406 samples taken from healthy people. In 2001–2009, the seroprevalence was estimated to be 4% (18 of 451). A total of 337 individuals tested in 2009 were exposed to at least one mosquito exposure-related risk factor. Within this group, 5% were seropositive for WNV. Most of the people with IgG positive against WNV did not have screen protections on windows and doors of their houses, while only 0.9% of those using window screens were seropositive for WNV [94]. During the same period, 56,757 mosquito specimens sampled on migratory and domestic bird reservoirs, were all negative for WNV RNA. A serological analysis by ELISA based on WNV recombinant envelope E (rE) protein and PRNT showed, for the first time in Serbia, that 12% of 349 horses from the northern part of country sampled in 2009–2010 presented specific neutralizing WNV antibodies [95]. Due to the absence of routine diagnosis and the limited resources of hospitals in Serbia, human cases of meningoencephalitis of unknown origin were not tested until 2012. In addition, regular surveillances of sentinel chicken, horses or mosquitoes are not performed. Consequently, the approach used to search for the virus in Serbia had been focused on detection of IgG-positive humans and virus in field-collected mosquitoes. In 2010, WNV linage 2 was detected in *Cx. pipiens* [94]. In August 2012, an outbreak of WN in humans was reported for the first time in Serbia (http://www.episouthnetwork.org/content/episouth-weekly-epi-bulletin-e-web; http://ecdc.europa.eu/en/healthtopics/west_nile_fever/West-Nile-fever-maps/Pages/index.aspx.). During the same year, viral RNA was detected for the first time in nine wild birds. All these isolates belonged to the WNV lineage 2 and were closely related to strains responsible for recent outbreaks in Greece, Italy and Hungary [96]. From 2005 to 2013, WNV surveillance activities in Vojvodina province, northern Serbia, were performed as part of ongoing research projects. In 2014, a specific and integrated surveillance system targeting mosquitoes [97], wild and sentinel birds as well as horses, was set up by the National Veterinary Directorate in Vojvodina. The main goals of this nationwide WNV surveillance have been to provide warnings of WNV circulation and evidence-based tools for controlling the spread of WNV infections in humans.

### Tunisia

Since the first half of last century, rapid urbanization and changes in agriculture practices have created environmental conditions favorable to the proliferation of *Cx. perexiguus* and *Cx. pipiens*. The latter became the dominant species in urban and rural areas. Consequently, WN became the most important arboviral disease for public health. In Tunisia, the first outbreak of meningoencephalitis due to WNV was observed in autumn 1997 in two coastal districts (Sousse and Sfax) causing more than 173 cases with 8 deaths [98]. The WNV belonged to the lineage 1a (Tunisian strain PaH001) which is closely related to the group of American/Israeli viruses collected between 1998 and 2000 [99]. The second WN outbreak was reported in 2003 in all East coastal districts. Twenty patients with neurological signs including three fatal cases were reported in the district of Sousse [100]. In 2007, a total of 1854 sera collected from healthy patients from three different districts (north, center and south) were investigated by ELISA to detect specific IgG against WNV. Specific IgG were detected in 12.5% of studied population. The seroprevalence varied largely between the three districts: high endemicity in the center (27.7% in Kairouan district), moderate in the southern region (7.5% in the Sfax district), and low in the north (0.7% in the Bizerte district) [101]. The third important outbreak of WN was observed in 2012; 86 cases with neurological signs were confirmed by serological tests, 12 patients died [102]. Equids are also affected: the serological investigation tested by competitive enzyme-linked immunoassay of 284 horses conducted in 2012 in the southern west region of the country showed that 120 (42.3%) had WNV-specific neutralizing antibodies. The prevalence was significantly higher in areas close to the oasis compared with that of the surrounding arid areas [103].

Sandfly fever viruses belonging to the *Phlebovirus* genus of the *Bunyaviridae* family were occasionally reported. They include two main serocomplexes: Naples (Sandfly fever Naples virus, SFNV) and Sicilian (Sandfly fever Sicilian virus, SFSV), which are associated with human diseases. Besides, Toscana virus (TOSV) is a variant of Sandfly fever Naples virus. It is endemic in Mediterranean countries especially in Tunisia. In a recent study, Fezaa et al. [104] showed that out of 263 patients with neurological disorder tested using ELISA, 12.2% (*n* = 32/263) were IgM positive for TOSV. Of these 32 patients, 78% (*n* = 25/32) were IgG positive. In addition, 12.8% (*n* = 18/140) of the cerebrospinal fluid (CSF) samples tested by RT-PCR were positive for TOSV. One CSF sample tested by RT-PCR revealed the presence of SFSV [104]. By RT-PCR, TOSV was detected in *Phlebotomus perniciosus* and *Phlebotomus perfiliewi* collected in north Tunisia. In addition, the Punique virus was identified in *P. perniciosus* [104]. Among 494 healthy individuals from various regions of Tunisia tested by ELISA for anti-TOSV IgGs, 47 people (9.5%) were positive. Seroprevalence varied with bioclimatic regions and gender [104].

Usutu Virus (USUV), which is a “new” emerging *Flavivirus* antigenically close to WNV and also transmitted by *Culex* mosquitoes, has never been reported in humans in Tunisia. In a recent study, antibody titers against USUV were reported in 10 equines (among 284) [103]. In a serological investigation, Nabli et al. [105] recorded that 0.2% of human serum samples (*n* = 1406) from different regions of Tunisia were positive to the Sindbis virus [105].

### Turkey

Arbovirus screening in vertebrate hosts and vectors conducted in the last 15 years have revealed the activity of arboviruses transmitted by mosquitoes, sandflies, ticks and *Culicoides* [106–108]. Among mosquito-borne viruses, WNV is known to be present in Turkey since the 1970s [109]. After the detection of serological evidence of WNV in various animals (ass-mule, cattle, dog, horse and sheep) in eight provinces [110], human cases were detected [61]. The WNV Lineage 1 clade 1a was confirmed in both humans and horses [111]. Further studies corroborated the circulation of the virus in the East (ducks and horses) as well as South East (horses and sheep), South (horses) and West parts of the country (dogs and sheep) [112]. The virus was also detected in primary and secondary vectors *Cx. pipiens* s.s. and *Ae. caspius* samples in North West [113], in *Cx. quinquefasciatus* and *Cx. perexiguus* in the South [112]. These results also confirmed the presence of the most important vector of the virus worldwide, *Cx. quinquefasciatus* with DNA barcoding for the first time in the country [112, 114]. Established populations of the invasive *Ae. aegypti* and *Ae. albopictus* were recorded in the northeast of the country in 2015 [15••]. Due to the fact that these species are involved in the transmission of CHIKV, DENV, YFV, and ZIKV, they will become a public health concern. Data from blood donors in recent years have shown evidence for DENV exposure in Central Anatolia, thus its reemergence is probable [115]. Imported cases of CHIK also represent a threat [116].

On the other hand, some major and novel *Phlebovirus* serotypes are endemic in Turkey. Sandfly fever Sicilian virus (SFSV), Naples virus (SFNV), and Toscana virus (TOSV) are known to be present in the country [106, 117]. In addition, Sandfly fever Turkish virus (SFTV), a variant of SFSV was discovered and *Phlebotomus major* s.l. is detected as the best vector candidate so far [118, 119]. Last year, Adana virus, a novel *Phlebovirus* belonging to the Salehabad virus complex was identified, with high seroprevalences in dogs, goats, sheeps but a low seroprevalence in humans [53]. A recent study revealed that dogs are candidate reservoirs of TOSV in Turkey and co-infection of this virus with *Leishmania infantum* is detected [120]. To put that into perspective, co-infection of TOSV and WNV was also documented in a human case for the first time, causing enhanced pathogenicity with severe clinical outcomes [121].

Caused by biting midges, bluetongue virus (BTV) is one of the most important diseases of domestic livestock. It was recorded in 15 provinces of Turkey, on Western [108], North Western [122], Eastern [123], South Eastern [124], and Central Anatolia [125] throughout the years, sharing lineages 2, 4, 6, 9, 10, 13, and 16 with its neighboring countries [108].

Tick-borne encephalitis (TBE) and Crimean-Congo hemorrhagic fever (CCHF) had a significant impact in Turkey. Since 2002, there have been more than 9700 patients infected with CCHFV with around 5% overall mortality rate [107]. Ticks collected from migratory birds were found infected with CCHFV genotype 4, whereas CCHFV Europe I clade is known to be present in Central Anatolia [126, 127]. In the same region, a recent study has shown that clade I is also affecting the Anatolian wild sheep *Ovis gmelinii anatolica* [128]. The virus was also detected in the north-western part of the country, outside the endemic region for several tick species [129].

## Conclusions

Arboviruses, mosquito-borne viruses in particular, including DENV, CHIKV, and WNV, are becoming a global health issue, spreading beyond their natural range of distribution, mainly in sub-Saharan Africa. DENV and CHIKV have benefitted from increasing human mobility and the larger geographical distribution of the vectors *Ae. aegypti* and *Ae. albopictus* which expand their area of activity to new regions and continents including high-income countries. The introduction of these viruses in naive countries relies on long distance spread taking advantage of human travels. By contrast, the spread of WNV depends primarily on bird migration and local viral dynamics relying on activities of mosquito populations. Given this global threat, the surveillance system (animal, human and vectors) must be strengthened to prevent outbreaks and/or to ensure the early detection of a potential epidemic. These are the objectives of the MedilLabSecure project: strengthen the preparedness for arboviral diseases in countries of the Mediterranean and Black Sea regions that should be considered a unique episystem for emergence of vector-borne diseases in Europe.
